# Propositions or Principles of Medicine

**Published:** 1827-04

**Authors:** 


### MEDICO-CHIRURGICAL

### REVIEW.

VOL. X.] Analytical Series. [No. 28.

"Nec tibi quid liceat sed quid fecisse decebit
"Occurrat, mentemque domat respectus honesti."

VOL. VI.] APRIL 1, 1827. [No. 12.

[NEW SERIES.]

### I.

### PROPOSITIONS, OR PRINCIPLES OF MEDICINE.

By F. J. V. BROUSSAIS, M.D. &c. &c.

WE have often contended that Professor Broussais carried his theory rather too far, in placing the *original* seat of fever exclusively in the mucous membrane of the stomach and bowels; though we believe that both the symptoms and dissections would shew the said structures more frequently implicated in fever, than any other part of the body. This was the conclusion to which the late Dr. Beddoes came, after examining the records of medicine in all countries respecting the *post mortem* appearances in fever. But, be that as it may, we consider the PROPOSITIONS, or PRINCIPLES of MEDICINE which M. Broussais has prefixed to his examination of "medical doctrines, and systems of nosology," as by far the best code of aphorisms or PRINCIPIA MEDICINÆ, which have ever been offered to the public in so concentrated a form. They are more in accordance with the advances which have been made in pathology and therapeutics, than any which have preceded them—and, in fine, we think them extremely well worthy of record in this Journal, and highly deserving the serious attention of the physiologist, pathologist, and practitioner. They are not indeed free from error, as we shall endeavour to shew, in occasional comments; but they contain a vast store of valuable principles adapted to practice, and serving as a safe guide between the extremes of dogmatism and empiricism.

We now enter on our task without farther preface, only premising that we shall occasionally somewhat condense the language without altering the sense of the propositions of Broussais. This article may be considered, therefore, as a correct translation of the new physiological doctrine.

### SECT. I.—PHYSIOLOGY.

1. Animal life can only be supported by stimulation from without;—and every thing which *augments* the vital phenomena is a stimulant.

2. Caloric is the primary and most important of all stimulants; and when it ceases to excite the animal economy, the others soon lose their action on the system.

3, 4, 5. (Relate to caloric.)

6. The composition of the solids and fluids is an ANIMAL CHEMISTRY; and the power which sets this chemistry in action, confers on the organs the faculties of feeling, and motion, by contraction. Sensibility and contractility, then, are the signs or proofs of life.

7. Certain substances in nature, besides caloric, augment the sensibility and contractility of those parts of the living body, with which they come in contact. This is stimulation or irritation, and the agents are stimulants.

8. Sensibility and contractility being augmented in one point, are soon augmented in several other points—this is sympathy.

9. Sympathy takes place through the medium of the nervous system.

10. All the phenomena of *association* take place through the medium of the nervous system, which transmits the stimulation from one part to several others—these also are sympathies.

11. The object and end of primary and sympathetic stimulation, are always NUTRITION, THE REMOVAL OF NOXIOUS CAUSES, AND REPRODUCTION. The movements by which these processes are accomplished are called FUNCTIONS:—but for the exercise of these functions the fluids must concur with the solids—in all stimulation there is an afflux or attraction of fluids to the part stimulated.

12. Sensibility and contractility are distributed in different degrees in the various tissues of the living body. Those which possess it in the highest degree, receive direct impressions from stimulants, and transmit the resulting action to the other parts: they are therefore the primary and natural movers of sympathies.

13. These tissues are particularly those on which the nervous matter is spread in the form of a pulp intermingled with the capillary vessels, sanguiferous and seriferous:—as the skin, the organs of sense, and the mucous membranes, which are the internal organs of sense.*

14. All the organs of sense, above-mentioned, are subjected naturally to the action of agents, both from without and from within;—and the stimulation or impressions which they receive are transmitted to the brain, their common centre, as well as to various other tissues and parts, by which the functions are sustained.

15. All stimulation capable of exciting a perception in the brain, traverses first the nervous system of relation; it is then repeated in the mucous membranes, whence it is again transmitted to the brain, which judges according to the counsel of the viscus to which the mucous membrane belongs, and acts according to the pleasure or pain experienced. This action has always for object the duration or repetition of the impression, or the removal of its cause.

16. The action determined by the cerebral centre of relation is executed through the medium of the loco-motive muscles—the same nerves which transmitted the impression to the brain, serving for the conveyance of the will to the muscles.†

17. While an impression, or rather the stimulation which results from an impression, traverses the nervous system of the viscera, it determines certain movements in the fibrous structure, making part of the viscera; modifies the circulation of all the fluids passing through them; and produces also involuntary contractions of the loco-motive muscles (if any) belonging to the viscus.

18. Whilst the stimulant influence of the brain is exercised, voluntarily or involuntarily, on the loco-motive muscles of the body, the stimulation is also communicated (but involuntarily) to the fibrous and vascular tissues of the viscera—because the

---

* Broussais is hardly justifiable in placing the highest degree of *contractility* as well as sensibility in the above tissues. Sensibility they undoubtedly possess—but the highest degree of contractility resides in the muscular system, and which may be called into action sympathetically by the impressions on the said tissues.—*Rev.*

† Or rather a portion of the same nerve, as it is now pretty well ascertained, that each nerve is composed of two distinct parts, one for motion, and the other for sensation.—*Rev.*

nerves of relation are common to the loco-motive muscles and to the viscera.

19. The voluntary movements having placed the edible materials in contact with the organs of assimilation, these last operate upon them, each in its own way.

20. Assimilation is a phenomenon of the first order, and which cannot be explained by the action of sensibility and contractility:—It is evidently owing to the creative power (puissance creative) and is one of the acts of the "living chemistry."

21. Absorption depends, in the first place, on the affinities of this "living chemistry;"—in the second place, on the exercise of the sensibility and contractility.

22, 23, 24, 25, relate to the circulation, nutrition, excretion, and reproduction, which we may pass over.

26. States the existence of the ganglionic nerves, having the ganglia of the abdomen for their centre or sensorium.

27. The ganglionic nerves penetrate into the viscera and muscles, along with their vessels and their nerves of relation—many penetrate into the viscera and into the muscles of the trunk—few into the muscles of the extremities.

28. Injury of the ganglionic nerves does not cause, primarily, either pain or convulsions—*they do not transmit sensations to the brain, nor orders from the brain to the viscera.*

29. The ganglionic nerves, then, preside only over the interior movements or functions of the viscera, which are independent of the brain—they are grand agents, therefore, in the "living chemistry."

30. The ganglionic nerves receive and collect the stimulant influence of the cerebral nerves, over which supply the individual has no voluntary control, either of acceleration or retardation.

31. The ganglionic nerves, then, avail themselves of the vital force of the animal, for the purpose of this living chemistry, independently of any voluntary influence or power. When the sum of this vital force is not sufficient for the two great orders of functions, (animal and vital) the former is deprived of a portion, in order that it may be concentrated on the vital viscera. The ganglionic nerves appear to effect this diversion by cumulating the vital force, and with it the fluids, in the vessels of the viscera, and especially in the vessels of the brain—which is the cause of sleep.*

---

* We see this exemplified every day. Thus, if weakly people take a full meal, the vital force is not sufficient to supply the voluntary muscles, the intellectual operations, and the process of digestion. The two former func-

32. When irritation predominates in the viscera, the ganglionic nerves cause a reflux of it into the apparatus of relation, through the medium of the cerebral nerves, with which they are in intimate communication in the viscera; nor is it in the power of the will to prevent this reflux of irritation into the system of relation, no more than to prevent its ingress into the viscera from the aforesaid system at other times.†

33. The centre of relation, (the sensorium) solicited by the influence of the viscera, excites, with or without the concurrence of the will—with or without the consciousness of the individual, certain movements in the loco-motive apparatus, which are in direct ratio to the visceral irritations, and which cease only with the cessation of these irritations, or the engorgement and destruction of the brain itself.

34. Every time that there is excited in the animal economy a stimulation capable of giving a shock to the cerebral nerves of relation, this stimulation is transmitted to the sensorium, which may execute movements with or without the consciousness—with or without the will of the individual. The phenomena, therefore, of *consciousness* are not always present when perception and re-action of the sensorium are going forward.

35. The perceptions of the sensorium, accompanied by *consciousness*, bear the name of *sensibility*, and the movements which the sensorium directs with consciousness are called *voluntary* movements; but the perceptions of the sensorium, *unaccompanied* by consciousness, and the movements directed by the sensorium without the participation of the mind, cannot be attributed either to sensibility or voluntarity. They are a peculiar species of *organic* or automatic phenomena. The cerebral nervous system, therefore, exhibits two *modes* in its functions.‡

36. Every time the mind has a perception, it feels, at the same time, in the brain, and in some other part external to the brain. (Prop. 12.) But the extra-cerebral points of sensation are not confined solely to the external and internal organs of

---

tions, therefore, are more scantily supplied, and the vital force is directed to the digestive apparatus. Hence the powers of the mind are enfeebled—the muscles are unequal to exertion—and the individual has an inclination to lie down and sleep.

† This, too, we often see exemplified. Thus, when indigestible substances irritate the mucous membranes, or, in other words, the ganglionic system, there is a reflux of the irritation upon the muscles, and even upon the intellectual organs—hence the restlessness, fidgets, and cramps, which people experience at night, together with want of sleep, after too heavy dinners or suppers.

‡ Thus, worms in the intestines will cause irritation there, without the individual being aware of it, which irritation will be communicated to the sense; (described in prop. 12.) They are extended also to accidental foci of inflammation; for inflammation places the nerves of most tissues in a condition nearly analogous to that of the nerves of naturally sensitive surfaces. These foci of inflammation, therefore, become, as it were, accidental and temporary organs of sense.

37. The mind is capable of refusing to execute certain actions, in obedience to sensations excited by the natural and accidental organs of sense. (36.) But there are other actions, which it can only prevent for a longer or shorter period.

38. The mind has the power of retarding or preventing the execution of acts demanded by the sensations, *only* when the encephalic apparatus is in an advanced state of development—when it is in a waking state—and in health. This power or faculty is null in infancy—increases with the exercise of the intellect—and presents, in insanity and other morbid conditions, numerous modifications.

39. Those acts which the mind can only retard for a time, are such as are solicited by the sensations that proceed from the viscera essential to life, and which are connected with the urgent execution of their functions.

40. Among the acts which the individual can refuse to execute, there are *some* which are solicited by the wants of the vital viscera, but which may not be very urgent; (the taking of food, for example)—if they become urgent, the will must obey the demand, or alienation of mind, or death of the body, must ensue. The *other* acts which the mind can refuse to execute relate to functions which are not essential to the conservation of life; but the refusal, even of these, may produce insanity.

41. When the animal suffers and dies, from refusal to satisfy the demands of the viscera, it is the triumph of intellect over instinct. But when mental alienation is the consequence of this struggle, (from irritation of the brain) it is the triumph of instinct over intellect.

42. Instinct consists in sensations determined by the viscera,

---

sensorium, through the medium of the cerebral or spinal nerves distributed to the viscera, and there produce a sensation or impression, without our consciousness of such sensation—and the effect or consequence of this irritation will often be convulsions or irregular movements of the muscular system, quite independent of the will. This example explains one of the modes of function in the cerebral nervous system—a function which plays a most important part in numerous diseases, and which has tended, more than any other law of the animal economy, to puzzle the practitioner, and lead into error.—*Rev.*

and which solicit the cerebral centre to execute acts necessary for the exercise of their functions.

43. The acts solicited by instinct are often executed without the participation of the will, and even without consciousness. Examples are found in the fœtus, and during sleep.

44. The acts solicited by the instinct predominate in the infant, and diminish in proportion as the intellect becomes perfected.

45. The intellect manifests its influence on the organism by the modifications which it impresses on the sensations determined by the instinct, and on the acts solicited by the latter.

46. The passions are sensations at first excited by the instinct; but afterwards fomented and increased by the attention bestowed on them by the intellect, so as to become predominant, and leading to acts more or less remarkable, but always directed to the gratification of the instinctive wants, which were their origin.

47. The passions are, like insanity, the triumph of the viscera, and consequently of instinct, over intelligence. In this way they often lead to mental derangement.

48. There is always a mixture of instinct and intellect in the passions.

49. Instinct may be exercised with or without the intellectual faculties.

50. The intellectual faculties have always some portion of instinct mixed up with them.

51. The intellectual faculties may be exercised without passion; but never without a mixture of pleasure or pain.

52. The pleasure and pain which accompany the exercise of the intellectual faculties have the same seat as the pleasure and pain of the passions—for the sensorium cannot feel in the brain without feeling also in the viscera—and it is in these last that it feels most.

53. When the intellect is occupied with ideas relative to the wants of a viscus, or the functions of an organ of sense, the nerves of this viscus or this organ of sense are always in action, and transmitting sensations to the sensorium; hence it results that the destruction of a nerve of sense causes, by degrees, an abolition of the ideas that used to be produced through this medium.

54. An acephalous fœtus may live till its separation from the mother, when it dies, because the respiration depends on the brain.

55. Organs whose communication with the brain is completely cut off, soon lose their vitality. But this is rare; for in paralysis, the consequence of affection of the brain, there is still some communication left, and the members live, though in an imperfect condition.

56. It is not from the defect of a peculiar principle derived from the brain that paralysed parts waste, but from want of excitation and exercise.

57-58 relate to paralysis.

59. A free and constant communication of excitement between the brain and all parts of the body is indispensible for the maintenance of an equilibrium in the functions of the body.

60. In hot climates and in hot seasons, excitation is more produced from the exterior than from the interior surfaces of animals, and *vice versa* in cold climates and cold seasons. In these latter circumstances, the gastric surface becomes the principal medium of excitation, and nutrition is then more active.

61. Excitation is never uniform in the animal economy:—it is always *plus* in some parts, and *minus* in one or several other parts—thus predominating successively in various regions of the body. This inequality often goes so far as to produce derangement in the equilibrium of the functions.

62. Health is never impaired spontaneously; but always because the external stimuli, destined to sustain the functions, have accumulated excitement in some part—or because they have been insufficient for the animal economy—or because the animal economy has been stimulated in a manner which is repugnant to the laws of vitality.

63. There are certain external agents or modifiers (modificateurs) which diminish the phenomena of life in those organs to which they are directed; but the pain developed in the enfeebled part performs the office of an excitant, and recals the vital phenomena, sometimes in a salutary manner, sometimes in a manner unfavourable to the conservation of life.

64. Sanguification in excess augments the sum total of vitality; but this process has a boundary, beyond which, the excitation accumulates in some organ, and disease is the consequence.

65. Excitement may accumulate, or superabound in some organs or parts, although the sum total of excitement in the system may be below par! and this inequilibrium may go on to marasmus or even death.

66. The animal economy can never long sustain super-irritation, or immoderate stimulation with impunity. Those who appear to be completely habituated to inordinate excitants, sooner or later experience the consequences, in the shape of local disease or super-irritation.

Here terminate the propositions of physiology; and we are inclined to think that they well merit the attention of the medical practitioner. They contain many profound views of the animal economy in health—views which naturally lead to others still more profound and important in disease. We shall therefore, proceed at once to the second division of the subject.

### SECT. II.—PATHOLOGY.

67. Health consists in regularity of the various functions—disease in their irregularity—death in their cessation.

68. The functions are irregular when one or more are exercised with two much or too little energy.

69. The energy of a function is in excess, when it precipitates, suspends, or deranges other functions with which it is associated, so as to menace the organs on which these functions depend.

70. The energy of a function languishes, when the organ or organs by which it is performed do not enjoy a sufficient degree of vital power.

71. The vitality of an organ may have been too much exalted before it became diminished, and *vice versa*.

72. There is no such thing as a *general and uniform* diminution or exaltation of the vitality of all organs.

73. Exaltation of the vital force always commences in one organic system, and is thence propagated to one or more other systems.

74. The nature of the exaltation thus communicated from one organ to another, is the same as the original exaltation—namely, an augmentation of the vital phenomena.

75. The exaltation of one or more organic systems invariably occasions languor or depression in some other system or apparatus.

76. The diminution of vitality in one or more organs *often* occasions exaltation of one or more other organs or parts—but *sometimes* a diminution in these last.

77. The exaltation of vitality in an organ or apparatus—presupposes a degree of excitement produced there by stimulants, which is incompatible with the maintenance of health—that is to say, a super-excitation produced by super-stimulation.

78. A local or partial super-excitement always supposes a too great afflux of fluids to the parts, viz.—a *congestion* prejudicial to the exercise of the functions of the part.

79. The union of super-excitation and partial morbid congestion always induces an unnatural or irregular nutrition of the part—thus constituting a state of *active congestion,* necessarily tending to disorganization.

80. Super-excitation and active *local* congestion are compatible with a *general* diminution of the sum total of vital force in the individual.

81. Local diminution of vital force is always followed by local diminution of nutrition, though *passive* congestion may sometimes co-exist in the part.

82. Passive congestion may cause disorganization, but this not nearly so often as active congestion.

83. Active morbid congestion being always the companion of super-excitation or *super-irritation,* this last word will be used, for brevity's sake, as comprehending the said condition of *active morbid congestion ;* and this is to be particularly borne in mind.

84. Irritation may exist in a system or apparatus, without any other system or apparatus being involved; but then the irritation is very inconsiderable. As soon as the irritation rises to a certain point, it is propagated to other systems or parts, more or less remote, and that without any change in its nature.

85. The nerves are the sole agents in the transmission of irritation—thus constituting the MORBID SYMPATHIES, which are effected in the same way as healthy sympathies, except that, in the *former* case, the nerves transmit irritation or super-excitation instead of the healthy quantity of excitation.

86. MORBID SYMPATHIES are of two kinds—organic sympathies, and sympathies of relation. The *former* are manifested by organic phenomena, as increased action, congestions, derangements in the secretions, exhalations, &c. changes in the temperature of the body, and vices of nutrition :—these are ORGANIC sympathies. The sympathies of RELATION are manifested by pains, convulsions of the voluntary muscles, and mental aberrations.

87. The organic sympathies may exist without the sympathies of relation; but these last induce sooner or later the former. In general, the two kinds of morbid sympathies co-exist.

88. The greater the sensibility of the organ that is irritated, and of the individual generally, the more the morbid sympathies are multiplied, and *vice versa.*

89. The more numerous and active the morbid sympathies, the more grave the malady.

90. Sympathies of relation (86) when in excess, may occasion death, which seems to depend on some serious derangement of the sensorium, in such cases. Excess in the organic sympathies may also cause death, by congestion and disorganization of one or more of the viscera.

91. The organ which is primarily irritated is sometimes the only one to suffer congestion or disorganization—the sympathising organs not being sufficiently irritated to partake in the congestion or disorganization.

92. The organs sympathetically irritated may contract a higher degree of irritation than that of the organ originally affected. In such cases the disease changes its place and name. This is metastasis.*

93. The organ which has become the seat of a metastasis, excites, in its turn, other sympathies proper to the organ—and these again may become predominant:—such are the ERRATIC INFLAMMATIONS, &c.

94. If the sympathetic irritations which the principal viscera determine in the organs of secretion, exhalation, and in the surface of the body, become more powerful than those in the viscera themselves, these viscera are relieved and the disease speedily terminates in a favourable manner. These are the CRISES. In such cases, the irritation proceeds from the interior to the exterior.

95. Critical congestions always terminate by some evacuation from the system, whether secretory, purulent, or hæmorrhagic. Without one of these, the crisis is not complete.

96. If irritation proceeds from the exterior to the interior—or from one viscus to another of more importance, the disease is aggravated. These are the false crises of authors.

97. Irritations have no fixed duration or march; both one and other are determined by the idiosyncrasy of the individual, or by the influence of various external or internal agencies.

98. Irritation has a tendency to propagate itself by similarity of tissue and organic system—this is what constitutes diathesis. Nevertheless, it sometimes passes into tissues of a very different kind from those in which it originated—and this more frequently in acute than in chronic diseases.

99. When irritation accumulates the blood in a tissue, so as to present swelling, redness, and heat, capable of *disorganizing*† the part irritated, we give it the name of inflammation.

---

* This, we think, is the most simple, natural, and just explanation that has ever been given of metastasis, about which so many absurd controversies have taken place.

† By this is evidently meant a change from the natural or healthy structure, and not such a degree of change as is incompatible with return to healthy structure. This last acceptation is that in which we generally receive the word *disorganization* in this country.

100. Inflammation, even of the most intense kind, is not necessarily accompanied by local pain.

101. The local pain of inflammation offers a very great variety, dependent on the nature and degree of the sensibility of the part, and of the individual generally.

102. Inflammation often excites more pain in the parts which are sympathetically affected, than in those where the original focus of inflammation is seated. Inflammation of the mucous membrane of the stomach and bowels, &c. offers daily examples.*

103. When inflammation excites no pain, it only awakens the *organic* sympathies. (86.)

104. Inflammation always effects a change in the fluids of the part inflamed. 105. It may exist without suppuration.

106. Inflammation often leaves, as a sequela, a kind of irritation, which does not go by its own name, and produces a kind of *cacochymia*, which has been falsely considered as idiopathic.

107. Inflammation often excites sympathies of relation (86) which have occupied the attention of writers and practitioners, as idiopathic diseases, and are called *nervous*,—the neuroses.†

108. Inflammation does not change its nature in consequence of the debility which it induces.

109. The irritations of all organs are transmitted to the brain, where they acquire a certain degree of intensity, especially if they be inflammatory irritations. There is then induced an alteration of the intellectual and affective faculties, and a state of malaise or even pain, which is referred to various parts rather than to its primary seat. An excess in this morbid sympathy ends occasionally in encephalitis.

110. Intense irritations of all organs are constantly transmitted to the stomach, from the very beginning—hence result inappetence, alteration of colour in the tongue and its secretions, &c. If this irritation, when propagated to the stomach,

* The same may be said of *irritation*. This, though seated in the stomach or small intestines, will often shew itself any where and every where, rather than in the spot where it has its head quarters.

† Here the ingenious author begins to tread on tender ground, though not entirely untrodden before. Dr. Parry resolutely maintained that the *neuroses* were occasioned by *determination of blood*, which is nearly the same thing as what Broussais is contending for in this place. If, for the terms used by both authors, they had substituted *irritation*, we should have been ready to agree with them. As it is, the doctrine of Broussais is one of great interest, as will be seen in our progress. It will tend greatly to remove some of the obscurity which involves the class neuroses, and prove that many of them which are considered idiopathic, are only *sympathetic* irritations of disordered viscera or deranged secretions.

rises to the point of inflammation, we have then the symptoms of gastritis, in consequence of which, the brain is more irritated than before, and, in a very high degree of this sympathetic cerebral irritation, inflammation of the brain itself may take place.'

111. Intense irritations of all organs are transmitted to the heart; and then the circulation is accelerated, the heat augmented, and most unpleasant sensations excited on the surface. This is fever, taken in a general or abstracted point of view.

112. Fever is always the consequence of irritation of the heart, primitive or sympathetic.

113. Every irritation sufficiently intense to produce fever is a shade of inflammation.

114. Every inflammation sufficiently intense to produce fever, when propagated to the heart, is, at the same time, sufficiently so to be propagated to the brain and stomach—and as the nature of inflammation is not changed by its sympathetic propagation from one organ to another, the affection of heart, brain, and stomach, in these cases, is a grade or shade of inflammation.

115. Irritations transmitted to the brain and stomach from an inflamed organ, sometimes diminish, while the original inflammation persists; and these two organs (brain and stomach) regain their functions, whilst the heart continues to be greatly irritated, and to keep up the fever.

116. Although the brain and stomach continue their functions during the inflammation of other organs; yet they are nevertheless irritated *organically*. (86.) This irritation borders on inflammation, and not seldom changes into this state, if the original focus of inflammation continues till death.

117. If the irritation excited by sympathy in the stomach and brain, instead of diminishing, becomes more intense than that in the original focus—the case is metastasis of inflammation to these organs.

118. Inflammation of the encephalon *always* draws after it inflammation of the alimentary passages, and *sometimes* of the organs annexed to these passages:—this is an organic sympathy.*

119. Inflammation of the encephalon is more frequently the *sympathetic effect* of inflammation of the stomach, than the *cause*.

---

* We cannot agree with M. Broussais on this point, unless he modifies the term inflammation into *irritation*. With this modification it would be difficult for any close observer of nature to deny the truth of the proposition.

120. The sanguineous congestion of the stomach in ebriety, typhus, and other fevers *mali moris*, is propagated to the brain and also to its coverings.

121. Encephalic inflammation excites *nervous phenomena*, which are often considered erroneously as idiopathic affections.

122. All irritations of the encephalon, which are prolonged till death takes place, end in inflammation or hæmorrhage—such is the case in fatal epilepsy, catalepsy, and mental perturbation in excess.

123. Mania always supposes an irritation of the brain:—which irritation may be long kept up there by inflammation in a distant part, and subside when that inflammation subsides; but, if continued for a certain period, it generally ends in veritable encephalitis, parenchymatous or membranous.

124. No extra-cerebral inflammation can produce mania, until the stomach and small intestines become affected. In such cases the liver is affected secondarily.

125. Arachnitis is more frequently consecutive of gastro-enteritis than primary:—but delirium, insomnium, and convulsions, may be kept up by this gastro-enteritis—disappear as it disappears—or leave, after death, but very faint traces of its previous existence.

126. All severe suffering, whether from inflammation of an organ, stimulation of a branch of a nerve, or mental perturbation, tends to produce fulness of blood in the brain, (engorge le cerveau,) and develop inflammation there or in its coverings. But the sufferings of the stomach are the most cruel of all—and each and every of the above causes produce this gastric derangement. Gastro-enteritis never obtains without exciting more or less of cerebral irritation.*

127. Tubercles, cancers, and other organic diseases of the brain, are the products of chronic inflammation of that organ.

128. All irritations of the encephalon may border upon, if not end in, apoplexy.

129. The term APOPLEXY means the cessation of the phenomena of relation, (abolition of sense and motion.) We may distinguish two principal *degrees* of this disease by the absence or presence of paralysis; but no division can be properly founded on the pathological characters of the brain, as ascertained by dissection.

130. Inflammation of the internal or mucous membrane of

* There is great truth in this proposition—especially if we substitute irritation for inflammation. The quantum of mental misery produced by irritation in the stomach and bowels can only be estimated by the dyspeptic invalid!

the stomach, termed gastritis, can never be verified by dissection, but in connexion with inflammation of the internal membrane of the small intestines. It would be more proper, therefore, to give it the name of *gastro-enteritis.*

131. Inflammation of the internal membrane of the small intestines is sometimes seen in the dead body, without any corresponding state of the stomach; but this isolated enteritis can never be positively recognized in the living body—hence an additional reason for using the term *gastro-enteritis.*

132. Gastro-enteritis presents itself in two forms, marked by the predominance of the gastric or intestinal phlegmasia. The pain of stomach, the nausea, vomiting, and inability to bear nourishment, characterise the gastritis—the power of satisfying the thirst, the rapid absorption of fluids swallowed, are signs of the predominance of enteritis. The other symptoms are common to both affections, with very few exceptions.

133. Acute inflammation of the mucous membrane of the small intestines, without affection of the peritoneum, is not attended with colicky pain in the majority of cases—but, often with only a sense of heat or malaise, and constipation of the bowels. Invaginations of this part of the intestines seldom cause ileus, or even pain, except of a slight colicky nature.

134. Colick, diarrhœa, and tenesmus, are the proper signs of inflammation of the mucous membrane of the COLON. 135. *Colitis* is the proper term for this phlegmasia, which, however, often succeeds or accompanies *gastro-enteritis*, as above defined.

136. Gastro-enteritis is unaccompanied by any fixed pain, where the inflammation is not severe, and extending to the stomach or duodenum:—and even pressure on the abdomen does not call forth pain.

137. *Gastro-enteritis* is distinguished by the sympathies which it excites:—1mo, by organic sympathies, viz. redness and heat at the open extremities of the mucous membranes, (in the mouth for instance)—the same in the skin—derangement of the biliary, urinary, and mucous secretions. 2ndo, by sympathies of relation, viz. pains in the head and limbs—confusion of the feeling and judging faculties, (l'aberration de la faculté de sentir et de juger.) The influence exercised on the heart by this phlegmasia, is common to many other inflammations.

138. Acute gastro-enteritis, if exasperated, gives rise to stupor, fuligo, livor, fetor, prostration of strength, and, in short, to those phenomena described in putrid, adynamic, and typhoid fevers. If the sympathetic *irritation* of the brain passes into *inflammation*, we have delirium, convulsions, &c. exhibiting the characters of malignant, nervous, or ataxic fevers.

139. All the idiopathic (essential) fevers of authors, are referrible to simple or complicated cases of *gastro-enteritis*. This phlegmasia has been overlooked when unaccompanied by local pain; or the local pain, when manifested, has been considered as accidental.

140. Authors, indeed, have sometimes admitted that there were certain fevers dependent on inflammation of the digestive organs; but they have never maintained that what are called idiopathic fevers are solely dependent on this cause. It is only since the new physiological doctrine has been broached that this theory has obtained.

141. Authors being ignorant that the mucous membrane of the small intestines may be inflamed without causing local pain, have, one and all, attributed to *enteritis* the symptoms of *peritonitis*.

142. It is by acute gastro-enteritis, (always bear in mind that this term means inflammation of the *mucous membrane*, and not of the peritoneal coat) the first effect of the contagious virus, that small-pox makes its debut. Cutaneous inflammation supersedes the gastro-enteritis, and soon terminates, if the pustules be few in number; but if they are very numerous a severe cutaneous inflammation takes place, from the confluence of the pustules. This is the *secondary*, or, as it is called, the *suppurative fever* of small-pox.

143. It is by gastro-enteritic, catarrhal, guttural, or bronchial inflammation, that measles and scarlatina commence. It is in these phlegmasiæ that all the danger consists—namely, by their extending to the brain and viscera, in the sympathetic manner already described. The angina in scarlatina becomes often fatal; and the bronchial inflammation of measles is sometimes dangerous by passing into pneumonia, and interrupting the function of the lungs.

144. Hypochondriasis is the effect of a chronic gastro-enteritis acting sympathetically on a brain already disposed to irritation.*

---

* If for *gastro-enteritis*, M. Broussais substituted *gastro-intestinal irritation*, we would entirely concur with him in this proposition. In our present number will be found an interesting case of hypochondriacism, in the person of a late general officer, with the appearances on dissection. The disease lasted twenty years, and did not kill the patient at last, for he died suddenly of another disease. There was no vestige of chronic gastro-enteritis; but there was unequivocal proof of gastro-intestinal irritation for 20 years

145. Most cases of dyspepsia, gastrodynia, pyrosis, cardialgia, and bulimia, are the effects of chronic gastro-enteritis.*

146. Umbilical colics, of an intermittent or remittent character, with constipation, and without tenesmus, indicate certain shades of chronic inflammation of the mucous membrane of the small intestines, especially if they be unaccompanied by any symptoms of peritonitis. In general, this phlogosis is of an indolent and painless character.

147. The mesenteric glands do not inflame but as a consequence of *gastro-enteritis*—and this double phlegmasia constitutes *tabes mesenterica.* 148. These glands do not inflame from simple peritonitis.

149. Hepatitis is generally *consecutive* of gastro-enteritis, except when produced by external causes.†

150. Chronic gastro-enteritis is the cause of hepatic engorgements, and of those yellow and fatty enlargements of the liver, which we sometimes find in the bodies of those who die even of phthisis.

151. The dropsies of persons who have suffered from abuse of spirituous liquors, or inordinate drastic purgatives, &c. &c. are the effects of chronic gastro-enteritis invading the inner coats first, and slowly spreading to the peritoneal covering of the intestines.

152. Bulimia is the effect of a chronic gastro-enteritis, with predominance of irritation in the stomach and duodenum. This phlegmasia may exist in a degree that permits the assimilation of a quantity of aliment far greater than is necessary for the wants of the system—*hence result plethora, polysarcia, (obesity) and, sooner or later, the establishment of a great irritation in the brain, the joints, the kidneys, the heart, or, in short, on any point where accidental stimulation is more than commonly applied.*‡

153. These bulimial gastrites are often produced by the abuse of too stimulating a diet, and also by the abuse of medicines

---

previously to death. We refer the reader for an investigation of this subject to the second edition of Dr. Johnson's Essay on Morbid Sensibility of the Stomach and Bowels, page 58—69.

* The same remark applies to this proposition. Broussais should have used the term gastro-intestinal irritation. But it is to be remembered that this distinguished pathologist looks upon inflammation as merely a high grade of irritation; and this point being kept in view, we have little hesitation in agreeing with M. Broussais.

† This includes, or ought to include, climatorial influence, as intense solar heat, which experience has proved to be a powerful cause of hepatitis.

‡ In this term, M. Broussais appears to comprehend that voracious, or at least intemperate system of eating and drinking, which, sooner or later, called *stomachic*, administered when the gastric irritation is only in its incipient stages.

154. The exuberant assimilation and nutrition which take place in bulimial gastric affections, are always accompanied by more or less of local and sympathetic pains; which pains ultimately increase, so as to render the digestion uncomfortable and laborious, even when the appetite still continues in excess. This laborious and painful digestion ends in loss of relish for food, emaciation, nausea, vomiting, and all the thousand phenomena of dyspepsia. Sometimes acute gastritis supervenes on this state, when improperly treated by stimulants, irritating diet, &c.

155. When a long course of stimulating ingesta has greatly exalted the sensibility of the stomach, the treatment of dyspeptic affections is tedious and difficult. In these cases, it is rare for the sensorium to escape some degree of sympathetic irritation, and more or less of hypochondriacism is the consequence. Scirrhus or cancer of the pylorus occasionally terminates the scene.

156. Acute inflammation of the mucous membrane of the digestive apparatus often passes to the peritoneal covering, causing peritonitis.

157. Acute hepatitis is never mortal, unless the inflammation spreads to the mucous or peritoneal surfaces, the brain, or the thoracic organs.* 158. The same of nephritis.

159. The acute peritonitis of parturient women generally commences with inflammation of the uterus.

160. Long-continued irritations of the mucous membrane of the vagina very frequently produce inflammation of the cervix uteri, and also of the ovaries—hence scirrhus and cancer of the uterus, ovarian diseases, &c. 161. Scirrhus of the cervix uteri is often, however, the effect of violence suffered by this part during parturition.

---

terminates in gout, apoplexy, disease of the heart, dropsy, &c. With the latter part of the proposition, as marked in Italics, we perfectly agree with the talented author; but we have some difficulty in admitting that either *irritation* or *inflammation* in the mucous membrane of the stomach and duodenum can be the *cause* of the bulimial repletion :—We would be more inclined to consider them as the *effect* of such intemperance—because such effects almost invariably follow such modes of living, sooner or later.

* This proposition is imperfect, or rather erroneous. Acute hepatitis may end in suppuration destructive of the organ and of life, without involving the other *structures*, though, of course, their functions must be disordered in the last stage of any mortal disease. In hot climates, a complete disorganization of the liver is not an uncommon termination of inflammation, of which M. Broussais ought to have been aware.

162. Painful menstruation announces a permanent focus of irritation in the cervix uteri; and cancer of this organ is not an uncommon result, about the cessation of the catamenia, unless this irritation be removed long before that period.

163. Peripneumony often commences with catarrh or inflammation of the mucous membrane of the bronchia. The superior lobes of the lungs are then the principal seat of the inflammation. If this becomes chronic, tubercles are often developed in the summit of the parenchymatous structure, and phthisis follows.

164. Peripneumony of the middle and inferior portions of lung often commences without previous bronchial catarrh.—If it becomes chronic, tubercles will be developed, and phthisis will follow.

165. Pleurisy wastes the lung of that side by the pressure of the effused fluid, often without inflammation of that lung; but, in the mean time, pneumonia is frequently developed in the lung of the opposite side; and if this becomes chronic, phthisis will succeed.

166. Pleurisy predominating in the pulmonary pleura, without effusion or atrophy of the lung, sometimes induces inflammation of that lung, and may, by passing into a state of chronicity, develop tubercles.

167. The tubercles which succeed inflammation of the mucous membrane of the bronchia and air-cells are engendered in the same manner as enlargement of the mesenteric glands in chronic enteritis.

168. I have never seen pulmonary tubercles without previous inflammation. Even those which are found in the lungs of children, at birth, do not appear to be independent of this cause.

169. Tubercles form in all kinds of constitution, where chronic inflammation of the lungs or intestines has prevailed—but more readily in those who are of a scrofulous disposition, or, in other words, who are predisposed to irritations of the lymphatic system.

170. Cartilaginous, osseous, and calcareous depositions appear to be produced in the same manner as tubercles; and the same applies to melanosis, scirrhus, and various other morbid growths.

171. The term *phthisis pulmonalis* only expresses the disorganization which is the *product* of chronic inflammation of the pulmonary parenchyma, and, therefore, is a bad term for the disease: chronic pneumonia is a better word, specifying the tissue in which the inflammation commenced.

172. Inflammation often takes place in the serous covering of the heart, and is termed pericarditis. It is characterised by the seat of the pain, and by the depression and irregularity of the circulation, producing a sense of anguish in the region of the heart, lypothymia, and fear of death.

173. The heart also becomes inflamed through the medium of its internal or lining membrane, and the disease is then commonly called carditis. This phlegmasia affects in preference the arterial orifices, where it becomes chronic, and where it produces obstacles to the course of the blood, as thickenings, vegetations, ossification, &c. and ultimately hypertrophy of the heart, or aneurism. Irritation or inflammation of the locomotive system (rheumatism) is apt to be transferred to the heart, and give rise to this kind of disease.

174. Any irritation of the various tissues of the body, sufficiently intense to affect the function of the heart, may there produce phlogosis of either of the two membranes above-mentioned, and also of the internal surface of arteries.

175. Acute inflammation, ending in suppuration, of the muscular structure of the heart, is a very rare disease; but a wasting or softening of the muscular structure often takes place from long-continued inflammation of its membranes.

176. The worst effects of cardiac aneurisms are those which result from impediment to the circulation of the blood, as asthma, hæmorrhage, dropsy, &c. But *gastritis* seldom fails, in the end, to become associated with cardiac disease, especially if the treatment be stimulating.*

177-8. Ossification and dilatation of the aorta are often owing to chronic inflammation of the coats of the vessel. The same may be said of many other arteries in the body, when they become aneurismal.

179-80. Scrofulous affections are *irritations* of those exterior tissues of the body where the albuminous portion of the blood abounds; and as redness and heat are very little developed in such cases, the term inflammation is hardly proper. Should we not call them sub-inflammations? Real inflammation is not seldom superinduced on this state, or accompanies it through its whole course.

181. * * * 182. The skin is susceptible of chronic irritation. which is specially determined on the excretory apparatus of that

* We believe there is much truth in this. There is not a more constant symptom attendant on enlargement and other diseases of the heart than dyspepsia, and great irritation in the stomach. This part of the complaint every day masks the original malady; and even when the disease of the heart can no longer be overlooked, it is too often considered as sympathetic of the gastric disorder. Of this we have seen many deplorable examples.

tissue, and on the absorbents, changing the structure and disturbing the functions of the cutaneous surface.

183. The lymphatic glands are never enlarged, hardened, or softened, but in consequence of a species of irritation, which we may denominate sub-inflammation.

184-5. * * * 186. The cellular membrane or tissue, next after the mucous membranes, is the most susceptible of acute inflammation. Suppuration often takes place in these parts before the inflammation which precedes it is manifested by external signs.

187. Deep-seated abscesses, with resorption of pus, keep not up hectic fever, except by the irritation which is communicated to the different viscera, either by sympathy, or by the irritating impression of the resorbed pus. This fever, therefore, is not idiopathic.

188. Slow and indolent engorgements of the cellular tissue, even when the phenomena of inflammation are absent, are owing to an *exaltation* of their irritability (sub-inflammation)—and never to an opposite state.

190. When irritation has subsisted for some time, under the form of inflammation or sub-inflammation, in the membranes about the joints, the coats of arteries, and other parts not very extensible, there is an extravasation of albumen, and the fluid parts of this becoming absorbed, calcareous concretions are formed, as we see every day in gouty people. These concretions, then, are the *effects* of the previous irritation. 191. * * * Relates to melanosis, of which little is known.

192. External cancer, the product of irritative degeneration in the tissues where albumen and fat predominate, is always accompanied by inflammation. It is not to be considered incurable, as long as it is merely local.

193, 4, 5, relate to cancer, and are unimportant.

196. Inflammation of the serous membranes assumes two forms:—The one acute, very painful, and attended with much fever:—The other chronic, indolent, and nearly apyrectic. This last blends itself with the sub-inflammations.

197. Inflammation of the mucous membranes presents many more forms and degrees than that of the serous tissues; because, as the organs of internal senses, and the constant movers of various sympathies, the mucous surfaces are endowed with a sensibility and irritability infinitely more varied and intense than the serous membranes, which, in a state of health, possess neither sensibility nor sympathy.

198. All hæmorrhages not caused by external violence, and which are spontaneous, are *active*, whatever may be the degree of debility in the subject.*

199. Spontaneous hæmorrhages depend on an irritation of the sanguiferous capillaries:—The disposition is greatly increased by hypertrophy of the heart.

200. Spontaneous hæmorrhages have the same *remote* causes as the inflammations; hence we see hæmorrhage and inflammation complicated with one another—produce one another—and substituted for one another, in the same, or in different places.

201. The *neuroses* are active or passive, whilst the *phlogoses* are necessarily all active.

202. The *active neuroses* consist in exaltation of the sensibility in the nerves of relation, and of the muscular and vascular contractility, under the influence of these nerves. They are capable of affecting the loco-motive muscles, the viscera, and the capillaries, wherever the nerves of relation (cerebral and spinal) predominate. Examples, the neuralgiæ.

204. The *passive neuroses* consist in the diminution or abolition of sensibility and muscular contractility. They can only be complete in the loco-motive and sensitive apparatus.†

204. The active and passive neuroses acknowledge for cause, in most cases, an inflammation situated in the cerebral apparatus or in some of the viscera.‡ The passive neuroses depend sometimes on a sedative influence operating on the nerves of the part where the neurosis appears.

205. In the fixed active neuroses of the apparatus of relation (brain, nerves, muscles, and organs of sense) the capillary circulation is excited, and fulness of the vessels obtains. Inflammation or sub-inflammation exists or threatens to form in

* Dr. Parry came to nearly the same conclusion. He assigned as cause, an *increased* momentum of the blood. Broussais attributes it to local irritation, by which an afflux of blood is drawn to the part. The latter seems the more ingenious theory of the two; but we may demur against the *activity* of the hæmorrhage in all cases. Something must be allowed for the debility of the containing vessels, and the broken down state of the blood. Where these last obtain, as indicated by the symptoms and constitution, we see no reason why the term *passive* hæmorrhage should be blotted out of the nosological chart.

† Paralysis and loss of any sense exhibit examples of the passive neuroses.

‡ This is nearly the doctrine of the late Dr. Parry, who almost discarded the nervous system from his pathology, and attributed all nervous diseases to local determinations of blood. There is a very curious coincidence between these two great pathologists—certainly without any communication or knowledge of each other's sentiments. The doctrines of Broussais were as unknown to Parry, as those of Parry were to Broussais.

whatever tissue the neurosis is manifested, as well as at the origins of the nerves supplying the said tissues or parts. In the mean time the nervous trunks themselves are only concerned in transmitting the sympathetic influences from one point to another.

206. When, in the neuroses of the viscera of the chest or abdomen, there exist pain, or convulsions of the loco-motive muscles, there are two points of irritation, which are either in a state of inflammation or a condition that nearly borders on it —one point in the viscera, and the other in the encephalic apparatus.

207. Obstacles to the circulation do not derange the functions of the principal viscera, unless these obstacles are situated in the heart, or in the large vascular trunks.

208. In cases of obstacle to the circulation, dropsy results from the stasis of blood in the venous apparatus.

209. The sudden augmentation of dyspnœa in aneurism of the heart, from the exertion of locomotion, proves the influence of the muscular system on the venous circulation.

210, 211. Inflammatory congestions and also the secretions prove the influence of the capillary system on the circulation of the blood; while the process of absorption shews the influence of the capillary system or the progression of non-sanguineous fluids.

212. The malaise and anguish which attend material obstacles to the circulation, sooner or later determine gastric inflammation:—stimulating medicines accelerate the progress of this last affection.*

213. Scurvy is a peculiar condition of the solids and fluids, the product of imperfect assimilation, and having various causes, among which may be reckoned cold bad air, mental afflictions, and unwholesome aliments. Extravasation of the fluids is one of the most prominent phenomena of the scorbutic diathesis, because this malady renders the tissues fragile.† The viscera and the encephalic apparatus resist this extravasation longer than the other parts of the body.

---

* We have already alluded to this sympathetic affection of the stomach in diseases of the heart. The fact is certain, though the explanation is difficult, except on the principle of reciprocity of sympathy between the two organs.

† This, we think, is almost admitting the existence of *passive* hæmorrhage, notwithstanding the argument adduced in the next proposition, that inflammation may be complicated with, or even sometimes produce scorbutic extravasation.

214. The plegmasiæ readily associate with scurvy, but are not caused by it—on the contrary, they are often the cause of scurvy, as we daily see in the gums. 215. Unimportant.

216. Dropsy acknowledges for its physiological causes, obstacles to the course of the circulation, sanguineous and serous—chronic phlegmasia—diminished capillary action—imperfect assimilation—and debility.

217. Irritation presents natural intermissions in a state of health.*

218. *Morbid* irritation may also present an intermittent character in any organic apparatus or system of the body.

219. Morbid irritation may assume the *continued* form in an apparatus, in a moderate degree, with periodical exasperations, falling back, in the intervals, to its ordinary level. In these intervals, when moderate, it excites but few sympathies; but in its stage of exacerbation it develops a great number:—these are the *remittent* fevers of authors.

220. Intermittent and remittent irritations are always accompanied with exaltation of the sensibility and contractility, and consequently with congestion, as well in the principal seat of the irritation, as in those parts which sympathise with it.

221. These intermittent and remittent irritations are, in fact, phlegmasiæ, hæmorrhages, neuroses, or sub-inflammations, which exhaust themselves, and terminate by some critical metastasis or discharge. If they fail to terminate in this way, they become *continued* phlegmasiæ, hæmorrhages, neuroses, or sub-inflammations, acute or chronic.

222. Intermittent and remittent fevers are periodical *gastro-enterites*, in which the encephalon and the other viscera are sympathetically irritated, the same as in continued fever, and may become the principal seats of irritation or even inflammation, periodical or continued.

223. Each regular paroxysm of intermittent fever is a signal or sign of a gastro-enteritis, the irritation of which is transported to the surface, especially to the cutaneous exhalents, and thus a crisis of the paroxysm is produced. If the irritation is not

* We think it would have been more correct for M. Broussais to have said, "*excitement*, or rather excitability, presents natural intermissions, in health;" and we rather think that this is what he means, for in the next proposition he talks of *morbid* irritation presenting intermissions. In this country we consider *all* irritations as morbid—giving the term *excitement* to the healthy state.

See an interesting paper on intermittent irritations in the Periscope of this number.

thus completely dissipated, the fever is remittent;—if not dissipated at all, the fever takes the continued form.*

224. The masked, latent, disguised, or local fevers of authors, are periodical irritations of different systems or apparatuses, internal or external, but in which the heart is little influenced, and the general temperature but slightly exalted.

225. Those fevers which are called pernicious or malignant, do not differ from other fevers, except in violence, and in the danger of local congestions.

226. The dropsies which succeed intermittent fevers, always depend on one of the five causes or physiological modifications indicated in proposition 216.

227. The more ordinary external causes of intermittent fevers are atmospheric vicissitudes;—but every thing which acts on the animal economy in the way in which atmospheric vicissitudes act, may originate, and more especially reproduce these fevers.†

228. The cause of the periodicity of certain painful affections, and of certain convulsions, (as epilepsy) is not known.

229. Rheumatism is a phlegmasia of the fibrous and synovial structures, produced by atmospheric transitions; and it is not therefore surprising that it should sometimes take an intermittent or periodical character.

230. The articular periodical phlegmasiæ become wandering

---

* The doctrine of fever as here delineated, is, to say the least of it, extremely ingenious. Indeed, with a very slight modification, it stands a chance of being the most durable theory which has ever yet been constructed. There is a strong disposition in the professional world to view fever as almost invariably dependent on topical inflammation, though they are not agreed as to the locality of the phlogosis. It has been seen that, in M. Broussais' views, inflammation and irritation are only different grades of the same thing, and there can be little doubt that, in every fever, there is local irritation, in one or more organs. This narrows the question much; for we can readily conceive the existence of both a continued and intermittent irritation, though, when the term is extended to actual *inflammation*, the idea of periodicity is repugnant to our notions of pathology. The only other difficulty which we have in admitting M. Broussais' theory, is the *original* locality of the febrific irritation. The extensive chain of sympathies between the stomach and various other organs, and still more the intimate relation that subsists between the sensorium and the stomach, must render it extremely difficult to say where the irritation first commences. Upon the whole, the theory of M. Broussais, thus explained, and somewhat farther modified, appears to us extremely feasible, and we do not see any material objection that can be urged against it, in its application to practice, which is a consideration of great consequence.

† We much wonder that such an observer as M. Broussais should have passed over marsh exhalations, which are unquestionably the most powerful and frequent of all causes of intermittent fevers. These invisible agents we suppose he includes in the latter part of the proposition.

or migratory through the way of sympathy, and terminate by crises; or by fixing themselves on some particular part, in an acute or chronic form, in the same way as visceral phlegmasiæ do, when left to themselves.

231. Gout does not differ from common inflammation of articular structures, except in the circumstances attendant on the age, manner of living, and idiosyncrasy of the individual.

232. Unimportant.

233. That form of articular inflammation called GOUT, is often, but not always, complicated with a degree of chronic gastro-enteritis, which modifies its progress, and invites irritation towards the viscera.

234. The liver is only affected consecutively in gout, in consequence of the gastro-enteritis which accompanies it.

235. The irritation of gastro-enteritis is communicated to the articulations through the channel of sympathy, in the form of arthritis or gout; but this is only when the articulations have been predisposed to inflammation by atmospherical vicissitudes or other causes.

236. The irritation of the articular phlegmasiæ is propagated to the stomach sympathetically, and may there become the predominant affection occasionally.

237. The multiplied infirmities by which the aged subjects of gout are tormented, can only be attributed to the sympathies between the head, stomach, and other organs, which, in time, establish sub-inflammations, neuroses, or even phlegmasiæ of various organs and parts.

238. In chronic phlegmasiæ of the articulations, the irritation always tends to proceed from the circumference to the centre. This indeed is the case with all other inflammations of the periphery.

239. The transformation of gout into another malady is nothing more than a change of seat in the principal point of irritation—the phenomena being modified by the functions and structure of the part to which the irritation moves.

240. It is absurd to give the appellation of gout to an affection which has not been preceded by articular inflammation: for to say that gout is transferred to the brain, when mania supervenes on articular phlogosis, is as much as to affirm that mania is transported to the great toe, when an attack of gout supersedes an attack of mania.

241. In retrocessions of gout, it is of no other use to inquire what was the part inflamed previous to the metastasis, than to be able to determine the best part to which it may be recalled, by way of revulsion.

242. Revulsion is difficult, if not impossible, in cases of what is called "MISPLACED GOUT," except where the organ attacked is free from structural disease.

243. The narcotic vegetables, and alcoholic substances, in large doses, excite gastro-enteritis, (at first without ulceration) and engorge with blood the encephalic vessels, producing, in bad cases, convulsions. They also engorge the lungs.

244 to 253 relate to the action of various acrid and powerful substances on the stomach and bowels. They need not be detailed here.

254. Putrid animal substances which the stomach is unable to digest, produce gastro-enteritis, with irritation and engorgement of the brain, giving rise to the symptoms of typhus by the intensity of nervous phenomena. But ulceration of the mucous membrane does not take place, except consecutively, and after a certain duration of the inflammation. 255. Putrid fish and poisonous champignons produce the same effects.

256. All the irritating and escharotic vegetables, animals, and minerals, when applied to the skin in sufficient quantity, develop in the mucous membrane of the stomach and bowels, in the brain, and sometimes in the lungs, an inflammation analogous to that which is produced on the surface of the body, through the means of irritation transmitted from the periphery to the centre.

257. Poisons of every kind, if injected into the veins, occasion gastro-enteritis, if they are not sufficiently powerful to occasion death before the inflammation has time to form. 258. The same may be said of putrid animal substances or sanies inserted into the living flesh, or injected into the vessels. They produce the same effects as if taken into the stomach.

259. The bites or stings of venomous animals, where poison is left in the wound, determine a local phlegmasia, passing quickly into gangrene, according to the intensity of the irritation. The more virulent of these prove fatal by their deleterious influence on the nervous system. But if life continues long enough, the inflammation is repeated in the principal viscera, especially in the line of the primæ viæ, with a strong tendency to gangrene. The weaker poisons of this kind are restricted in their operation to the production of local inflammation.

260. The bites of mad animals determine also inflammation of the mucous surfaces, which is often repeated in the pharynx, brain, and even in the lungs. The delirium and convulsions appear to be consequences of these irritations and inflammations, varying according to the degree of susceptibility in the individual.

261. Worms in the bowels seem often to result from derangement of the intestinal secretions, and the increase of temperature attending inflammation of the mucous membrane, more or less acute.

END OF THE PATHOLOGICAL DIVISION.

We have now brought to a close the two sections on PHYSIOLOGY and PATHOLOGY, and hope we have done a service to the English profession, by presenting them with a more complete view of the doctrine of Broussais, or, as it is called, the NEW PHYSIOLOGICAL DOCTRINE, now so extensively prevailing on the continent. In our next Number we shall complete the subject, with the section on THERAPEUTICS, which is very extensive, and embraces a variety of the most important topics of discussion.

